# Zero Coronary Artery Calcium as a Marker of Vascular Resilience in High-Risk Adults

**DOI:** 10.1016/j.jacadv.2026.103031

**Published:** 2026-07-20

**Authors:** Olusola A. Orimoloye, Olufunmilayo H. Obisesan, Albert D. Osei, Omotola Oredipe, Mercedes R. Carnethon, Michael J. Blaha, Philip Greenland, Laura J. Rasmussen-Torvik

**Affiliations:** a Division of Cardiology, Northwestern University Feinberg School of Medicine, Chicago, Illinois, USA; b Department of Preventive Medicine, Northwestern University Feinberg School of Medicine, Chicago, Illinois, USA; c Section of Hospital Medicine, Department of Medicine, University of Chicago, Chicago, Illinois, USA; d Division of Cardiology, University of Pittsburgh Medical Center, Pittsburgh, Pennsylvania, USA; e Department of Internal Medicine, John H. Stroger Jr. Hospital of Cook County, Chicago, Illinois, USA; f Division of Cardiology and Ciccarone Center for the Prevention of Cardiovascular Disease, Johns Hopkins University School of Medicine, Baltimore, Maryland, USA

**Keywords:** atherosclerosis, cohort studies, coronary heart disease, primary prevention, risk stratification

## Abstract

**Background:**

Atherosclerotic cardiovascular disease (ASCVD) prevention relies on risk estimation. Yet among adults labeled high risk by the Pooled Cohort Equations (≥20%), coronary heart disease (CHD) event rates vary, with some experiencing few events, raising the possibility of a resilient phenotype.

**Objectives:**

This study aimed to investigate whether a coronary artery calcium (CAC) score of zero identifies a resilient lower-risk group among adults with high calculated ASCVD risk and compare their risk factor profiles with those with non-zero CAC.

**Methods:**

We analyzed participants in the Multi-Ethnic Study of Atherosclerosis with Pooled Cohort Equations–estimated ASCVD risk ≥20% and CAC data. Baseline characteristics were compared using standardized mean differences. Ten-year CHD events were analyzed using Kaplan–Meier methods, with HRs for CAC = 0 vs CAC >0 estimated using Cox models adjusted for age, sex, and race/ethnicity. Effect modification by age and sex was assessed.

**Results:**

Among 1,608 adults (mean age 73.2 years, 61.4% men) with ASCVD risk ≥20%, 359 (22.3%) had CAC = 0. Risk factors were similar between CAC groups, with standardized mean differences <0.20 for blood pressure, lipids, diabetes, and body mass index. Over 10 years, hard CHD incidence rates were 3.52 and 13.46 per 1,000 person-years for CAC = 0 and CAC >0, respectively. CAC = 0 was associated with lower CHD risk (HR: 0.27; 95% CI: 0.15-0.49). A significant CAC × age interaction (*P* = 0.023) indicated stronger protection at older ages.

**Conclusions:**

Nearly one-quarter of adults labeled high ASCVD risk had CAC = 0 and accrued few events despite similar risk factor burden. CAC = 0 may mark a resilient phenotype warranting targeted mechanistic investigation.

Traditional cardiovascular risk factors explain a substantial share of atherosclerotic cardiovascular disease (ASCVD) outcomes, including coronary heart disease (CHD) events, and have underpinned risk-based prevention frameworks since Framingham first defined the term “factors of risk.”^1^^,^^2^ Current risk estimation tools built on these risk factors inform contemporary guidelines that recommend the use of statins and other preventive therapies.^3^ Yet even robust models do not fully explain incident CHD or fully capture interindividual variability in disease expression.^4^ A recurring observation in clinical practice, clinical trials, and across cohorts is a discordance between estimated and observed risk, wherein some adults categorized as high risk per standard risk scores experience few coronary events.^5^

Coronary artery calcium (CAC) scoring, an anatomic measure of calcified atherosclerosis, has demonstrated the ability to go beyond standard risk scores and further distinguish individuals who are at risk for clinical events.^6^ In multiple studies, the absence of CAC (CAC = 0) is associated with very low near-term event rates, supporting its selective use to refine prevention decisions in the intermediate risk group, as is now reflected in the American College of Cardiology (ACC)/American Heart Association (AHA) guidelines for cardiovascular disease prevention.^3^^,^^7^^,^^8^

Separate from its utility as an advanced risk stratification tool, the absence of CAC in a minority of high ASCVD-risk individuals (based on the Pooled Cohort Equations [PCE])^9^ demonstrates that atherosclerosis is not an inevitable consequence of risk-factor exposure. These less common risk profiles strongly suggest the possibility of a biological phenomenon of resilience, where individuals maintain apparent vascular health despite substantial risk factor exposure. Prior MESA (Multi-Ethnic Study of Atherosclerosis) work showed that even among individuals with extreme risk factor burden (≥3 major risk factors), a meaningful proportion had CAC = 0 and experienced markedly lower short-term CHD risk.^10^ Parallel evidence from high-genetic-risk populations also supports this concept; in heterozygous familial hypercholesterolemia (FH), a fraction of individuals with CAC = 0 demonstrated very low near-term event risk despite lifelong high low-density lipoprotein (LDL) exposure.^11^

Despite these observations, “resilience” remains a descriptive idea rather than a well-defined phenotype. Accordingly, we sought to determine whether, among adults classified as high risk by conventional risk estimators, the absence of CAC identifies a resilient coronary phenotype characterized by lower observed risk of coronary events compared with high-risk peers with any detectable CAC. We further sought to define the risk factor profile of these individuals, quantifying the extent to which their traditional risk factor burden differed from those with CAC >0.

## Methods

### Study population

We analyzed data from the MESA, a community-based prospective cohort of 6,814 men and women aged 45 to 84 years who were free of clinically recognized cardiovascular disease at baseline. Participants were deliberately recruited to include 4 racial and ethnic groups (White, Black, Hispanic, and Chinese American) from 6 U.S. communities (Baltimore, Maryland; Chicago, Illinois; Forsyth County, North Carolina; Los Angeles County, California; Northern Manhattan, New York; and St. Paul, Minnesota) between July 2000 and August 2002. Institutional Review Boards at all participating centers approved the study, and all participants provided written informed consent. Additional design details have been published previously.^6^^,^^12^

For this analysis, we included Exam 1 participants with complete baseline data for CAC, PCE variables, relevant covariates (detailed below), and follow-up for “hard” CHD events defined as nonfatal myocardial infarction, resuscitated cardiac arrest, or CHD death in MESA. The analytic cohort was restricted a priori to individuals with estimated 10-year ASCVD risk ≥20% by the PCE.

### ASCVD risk estimation

Baseline 10-year ASCVD risk was calculated using the 2013 ACC/AHA PCE with sex- and race-specific coefficients. Inputs included age, sex, systolic blood pressure (and antihypertensive treatment), total and high-density lipoprotein cholesterol (HDL-C), current smoking, and diabetes, all captured at baseline.^13^ High ASCVD risk was defined as PCE risk ≥20% in concordance with current treatment guidelines.^3^

### Coronary artery calcium

CAC was measured at baseline using standardized, electrocardiogram-gated computed tomography protocols with a calibration phantom. Participants underwent duplicate scans interpreted at a core laboratory; Agatston scores were computed and averaged.^6^^,^^12^ Exposure groups were defined as CAC = 0 and CAC >0.

### Covariates and other characteristics

Baseline covariates were assessed at Exam 1. Sociodemographic characteristics (age, sex, race/ethnicity, educational attainment, household income) were collected via standardized questionnaires. Smoking status (never, former, current) and family history of CHD were assessed by self-report with standard instruments. Medication use was ascertained through a standardized medication inventory, in which participants brought all prescription and nonprescription medications for direct review and classification by trained study staff.^12^

Physical activity was measured with the Typical Week Physical Activity Survey. We summarized intentional exercise as metabolic equivalent minutes per week and secondarily categorized ≥500 metabolic equivalent minutes per week. Cumulative smoking exposure was summarized as pack-years.

Anthropometrics were obtained using standardized techniques; body mass index was calculated as kg/m^2^. Resting blood pressure was measured 3 times after a standardized rest; the mean of the last 2 readings was used. Hypertension was defined using the MESA-derived JNC VI variable (systolic blood pressure ≥140 mm Hg, diastolic blood pressure ≥90 mm Hg, or antihypertensive medication use). Estimated glomerular filtration rate (eGFR) was calculated using the Chronic Kidney Disease Epidemiology Collaboration equation.^14^

Fasting blood samples were processed per standardized protocols. Total and HDL cholesterol were directly measured; LDL cholesterol (LDL-C) was estimated using the Friedewald equation when applicable. Diabetes was defined as fasting plasma glucose ≥126 mg/dL, glucose-lowering medication use (including insulin), or self-reported physician diagnosis.

### CHD outcome ascertainment

Participants were contacted at regular intervals; events were adjudicated by physician committees using prespecified criteria. The primary endpoint was incident hard CHD, defined as nonfatal myocardial infarction, resuscitated cardiac arrest, or CHD death, with events centrally adjudicated by a committee of physicians using standardized criteria based on medical records, death certificates, and, when available, autopsy data.^12^ Follow-up accrued from baseline until first event, death, loss to follow-up, or administrative censoring. To align with the PCE horizon, primary survival analyses were administratively censored at 10 years.

### Statistical analysis

Baseline characteristics were summarized by CAC group using means (SD) or medians (IQR) for continuous variables and percentages for categorical variables. Between-group differences were quantified with standardized mean differences (SMDs); |SMD| ≥0.20 was considered potentially meaningful. For categorical variables, SMDs were calculated using pooled standardization of group-specific proportions.^15^

Time-to-event analyses focused on 10-year hard CHD events. We estimated cumulative incidence with Kaplan-Meier methods and compared groups with log-rank tests. Proportional hazards assumptions were evaluated using Schoenfeld residuals. We also calculated incidence rates (events per 1,000 person-years) within CAC groups and their absolute rate difference.

Cox proportional hazards models were used to estimate HRs and 95% CIs for CAC = 0 vs CAC >0 (reference), with robust variance estimators. To minimize conditioning on the risk factors that determined cohort eligibility (PCE ≥20%), the primary model (model 1) adjusted only for demographic variables: age, sex, and race/ethnicity. A fully adjusted model (model 2) additionally included systolic blood pressure, LDL-C, smoking status, diabetes status (normal, impaired fasting glucose, untreated diabetes mellitus, and treated diabetes mellitus), and family history of CHD and is reported in supplemental analyses (Supplemental Table 1).

Because age and sex distributions differed between CAC groups, we assessed effect modification by including interaction terms for CAC × age (age centered at 75 years) and CAC × sex in separate multivariable Cox models. Age-specific HRs at ages 70, 75, and 80 years were derived from the continuous interaction model using linear combinations of coefficients. Statistical significance of interaction was assessed with Wald tests for the cross-product terms using robust variance estimates.

Because the PCE predicts hard ASCVD events (myocardial infarction, CHD death, and stroke) rather than hard CHD alone, observed-to-expected (O:E) ratios for PCE calibration used hard ASCVD as the observed endpoint to match the prediction horizon. Expected events were calculated as the sum of individual PCE-predicted 10-year ASCVD probabilities within each CAC group. CIs for O:E ratios were derived using exact Poisson methods based on the observed event count. A supplementary O:E analysis restricted to hard CHD events was also performed.

To address competing risk from non-coronary death in this older cohort, we performed a sensitivity analysis using the Fine-Gray subdistribution hazard model,^16^ with non-CHD death treated as a competing event. The subdistribution HR was compared with the cause-specific HR from the primary Cox model.^16^

To assess whether demographic imbalances between CAC groups influenced the primary finding, we estimated propensity scores for CAC = 0 status using logistic regression with age, sex, and race/ethnicity as predictors, then restricted the analysis to participants within the 5th to 95th percentile of the CAC = 0 propensity score distribution.

As a sensitivity analysis, we repeated the primary analyses using the Predicting Risk of Cardiovascular Disease Events (PREVENT)-ASCVD equation^17^ with a ≥10% threshold to define the high-risk cohort, consistent with the 2026 ACC/AHA dyslipidemia guidelines.^18^ PREVENT-ASCVD scores were obtained from the MESA Coordinating Center. Both PREVENT (validated for ages 30-79) and PCE (validated for ages 40-79) were applied to all eligible participants regardless of age, including those aged ≥80 who are outside the validated range of both equations, to maintain consistency between the primary and sensitivity analyses.

As an additional sensitivity analysis, we repeated the primary models after excluding participants aged ≥80 years who are outside the validated range of both risk equations.^17^^,^^18^

In further sensitivity analysis, we repeated survival models after excluding participants using lipid-lowering therapy at baseline. Analyses used complete cases. All tests were 2-sided with α = 0.05. Statistical analyses were conducted in Stata MP, version 18 (StataCorp).

## Results

Of 6,814 adults in the MESA cohort, 1,608 participants with PCE-estimated 10-year ASCVD risk ≥20% and available CAC data were included in the analysis. The mean age was 73.2 ± 6.6 years, and 61.4% were men. At baseline, 359 participants (22.3%) had CAC = 0, and 1,249 (77.7%) had CAC >0.

### Baseline characteristics

Participants with CAC = 0 were younger (71.1 vs 73.8 years; SMD 0.40) and more often female (50.1% vs 64.7% male; SMD 0.30) compared with those with CAC >0. The prevalence of CAC = 0 within each age stratum declined with age: 36.1% of participants younger than 70 years, 20.4% aged 70 to 79 years, and 13.7% aged 80 years or older had CAC = 0. Racial and ethnic composition also differed, with 41.2% of the CAC = 0 group Black, 27.3% Hispanic, 19.2% White, and 12.3% Chinese, contrasting with the distribution in the CAC >0 group (Table 1).

**Table 1:** Baseline Characteristics of Adults With ASCVD Risk ≥20% Stratified by CAC Status (CAC = 0 vs CAC >0)

	CAC = 0 (n = 359)	CAC >0 (n = 1,249)	SMD
Sociodemographic variables			
Age (y)	71.1 (7.2)	73.8 (6.3)	**0.40**
Male (%)	49.9	64.7	**0.30**
Race/ethnicity (%)			
White	19.2	40.4	**0.48**
Black	41.2	25.7	**0.33**
Chinese	12.3	11.1	0.04
Hispanic	27.3	22.8	0.10
Education > high school (%)	49.6	53.6	0.08
Income > $50,000 (%)	20.9	24.9	0.10
Clinical risk factors			
Systolic blood pressure (mmHg)	145.7 (22.5)	143.3 (21.3)	0.11
Diastolic blood pressure (mmHg)	76.1 (11.4)	74.0 (10.4)	**0.20**
Total cholesterol (mg/dL)	190.8 (35.7)	193.3 (37.4)	0.07
Triglycerides (mg/dL)^a^	113 [76–165]	119 [84–171]	0.04
LDL-C (mg/dL)	113.8 (30.6)	117.5 (31.9)	0.12
HDL-C (mg/dL)	49.9 (14.2)	48.3 (14.2)	0.12
Lipoprotein(a) >50 mg/dL (%)	20.6	21.4	0.02
Fasting glucose (mg/dL)^a^	98 [89–121]	95 [87–112]	0.10
BMI (kg/m^2^)	28.7 (5.5)	28.0 (4.8)	0.13
eGFR, CKD-EPI (mL/min/1.73 m^2^)	72.2 (16.6)	68.7 (17.2)	**0.20**
hsCRP (mg/L)^a^	2.11 [0.96–4.05]	1.93 [0.90–3.99]	0.02
IL-6 (pg/mL)^a^	1.38 [0.88–2.03]	1.48 [1.01–2.18]	0.06
uACR (mg/g)^a^	9.5 [4.8–27.7]	9.5 [5.0–23.2]	0.09
Diabetes (%)	37.1	28.1	0.19
Hypertension (%)	81.9	76.6	0.13
Lipid-lowering therapy (%)	18.9	24.5	0.14
Family history of CHD (%)	37.1	47.3	**0.21**
Smoking status (%)			
Never	49.3	44.0	0.11
Former	34.3	41.9	0.16
Current	16.4	14.1	0.07
Pack-years^a^	0.05 [0.0–15.1]	2.6 [0.0–25.0]	**0.28**
Exercise ≥500 MET-min/wk (%)	57.3	61.4	0.08

Values are mean (SD) or median [IQR], as appropriate. **Bold** SMDs denote |SMD| ≥0.20, considered a meaningful between-group difference.

ASCVD = atherosclerotic cardiovascular disease; BMI = body mass index; CAC = coronary artery calcium; CHD = coronary heart disease; eGFR = estimated glomerular filtration rate by Chronic Kidney Disease Epidemiology Collaboration (CKD-EPI) equation; HDL-C = high-density lipoprotein cholesterol; hsCRP = high-sensitivity C-reactive protein; IL = interleukin; LDL-C = low-density lipoprotein cholesterol; MET = metabolic equivalent; SMD = standardized mean difference; uACR = urinary albumin-to-creatinine ratio.

a Medians due to skewed distributions.

Traditional risk factor burden was broadly similar by CAC status, with systolic blood pressure, LDL-C, HDL-C, total cholesterol, elevated lipoprotein (a) [Lp(a)], body mass index, diabetes, hypertension, and baseline lipid-lowering therapy all below the prespecified |SMD| ≥0.20 threshold for meaningful imbalance. eGFR (Chronic Kidney Disease Epidemiology Collaboration) was modestly higher in the CAC = 0 group (72.2 vs 68.7 mL/min/1.73 m^2^; SMD 0.20). Smoking status categories were comparable, although cumulative exposure (pack-years) was somewhat higher among participants with CAC >0 (SMD 0.28). Educational attainment and household income were similar (SMDs ≤0.10), and family history of CHD was modestly more prevalent with CAC >0 (SMD 0.21). Inflammatory markers (high-sensitivity C-reactive protein, interleukin-6) and urinary albumin-to-creatinine ratio were similar between groups (all SMDs <0.10) (Table 1).

### Association of CAC group with 10-year hard CHD events

**Figure 1:**
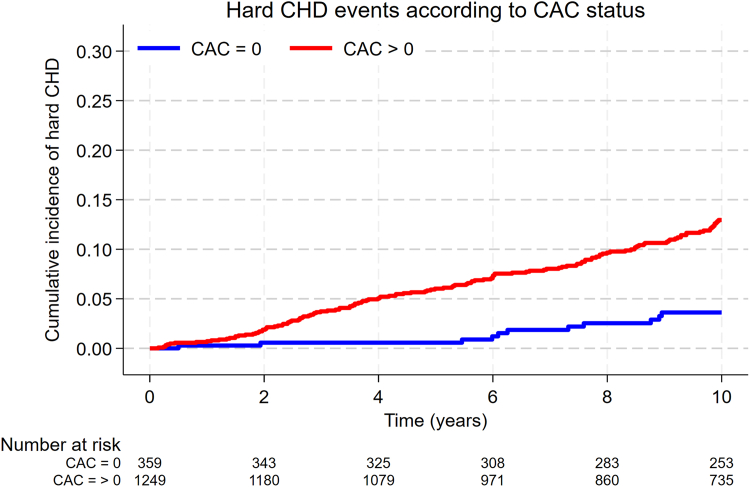
10-Year Cumulative Incidence of Hard CHD by CAC Status Kaplan-Meier cumulative incidence of hard CHD events (nonfatal myocardial infarction, resuscitated cardiac arrest, or CHD death) by CAC = 0 vs CAC >0 among adults with PCE-estimated ASCVD risk ≥20% in the MESA cohort (N = 1,608). Log-rank *P* < 0.001. ASCVD = atherosclerotic cardiovascular disease; CAC = coronary artery calcium; CHD = coronary heart disease; PCE = Pooled Cohort Equations.

Over 10 years of follow-up, 148 hard CHD events occurred. Event rates differed markedly: participants with CAC = 0 had 11 events over 3,128 person-years (3.52 per 1,000 person-years) compared with 137 events over 10,181 person-years among those with CAC >0 (13.46 per 1,000 person-years), an absolute rate difference of 9.94 per 1,000 person-years. Kaplan-Meier curves demonstrated clear separation of cumulative incidence by CAC group over the 10-year follow-up (log-rank *P* < 0.001; Figure 1).

In unadjusted analyses, CAC = 0 was associated with lower risk of hard CHD (HR: 0.26; 95% CI: 0.14-0.48). After adjustment for age, sex, and race/ethnicity (model 1), the association remained consistent (HR: 0.27; 95% CI: 0.15-0.49) (Table 2). The proportional hazards assumption was not violated (global Schoenfeld test, *P* = 0.17).

**Table 2:** Association of CAC = 0 (vs CAC >0) With 10-Year Hard CHD Events Among High ASCVD-Risk Adults

Model	HR (95% CI)
Unadjusted	0.26 (0.14-0.48)
Model 1 (age, sex, race/ethnicity)	0.27 (0.15-0.49)

HRs compare CAC = 0 vs CAC >0 (reference) for 10-year incident hard CHD, derived from Cox proportional hazards models with robust variance estimators. N = 1,608 (CAC = 0: 359; CAC >0: 1,249). Events: 148 (CAC = 0: 11; CAC >0: 137).

Abbreviations as in Table 1.

A statistically significant CAC × age interaction was observed (*P* = 0.023). Model-estimated HRs for CAC = 0 vs CAC >0 were 0.27 (95% CI: 0.14-0.51) at age 70, 0.18 (95% CI: 0.09-0.38) at age 75, and 0.13 (95% CI: 0.05-0.32) at age 80, suggesting that the protective association of CAC = 0 strengthens with advancing age. There was no evidence of effect modification by sex (CAC × sex interaction, *P* = 0.89).

### PCE calibration analysis

**Figure 2:**
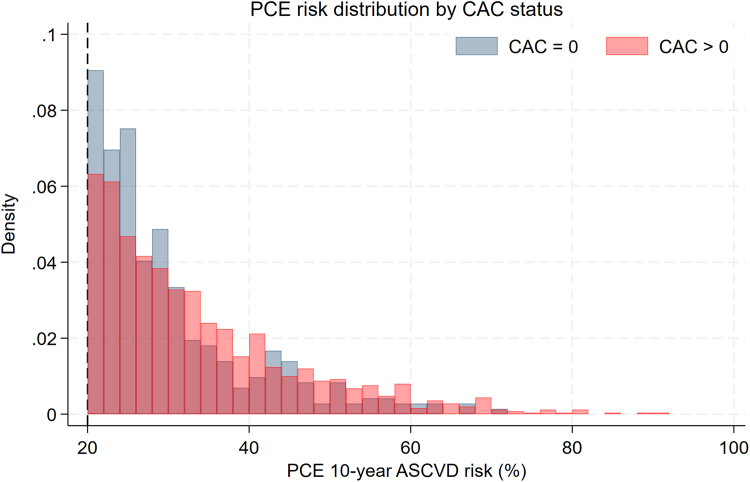
Distribution of PCE-Estimated ASCVD Risk by CAC Status Distribution of PCE-estimated 10-year ASCVD risk by CAC status among adults with PCE ≥20% (N = 1,608). Despite modestly lower median risk in the CAC = 0 group (26.7% vs 29.9%), the distributions overlap substantially. The majority of CAC = 0 participants had PCE risk well above the 20% inclusion threshold (60.4% ≥ 25%, 16.7% ≥ 40%). Abbreviations as in Figure 1.

Among CAC = 0 participants, the median PCE-estimated 10-year ASCVD risk was 26.7% (IQR: 22.8% to 33.3%), compared with 29.9% (IQR: 24.0% to 39.7%) in CAC >0 participants, with substantial overlap between groups (Figure 2). The majority of CAC = 0 participants were well above the 20% threshold: 60.4% had PCE ≥25%, 35.1% had PCE ≥30%, and 16.7% had PCE ≥40%.

To evaluate whether PCE miscalibration could account for the event deficit in CAC = 0, we computed O:E ratios for 10-year hard ASCVD events. In the CAC >0 group, the O:E ratio was 0.53 (95% CI: 0.46-0.60), consistent with the known overestimation of risk by the PCE. In the CAC = 0 group, the O:E ratio was 0.26 (95% CI: 0.17-0.37), with non-overlapping CIs. Since the PCE does not incorporate CAC and assigns similar predicted risks to both groups, uniform miscalibration would produce similar O:E ratios in both groups. The differential overestimation, approximately 2-fold in CAC >0 vs approximately 4-fold in CAC = 0, indicates an additional event deficit in CAC = 0 that PCE miscalibration alone cannot explain. A supplementary analysis restricted to hard CHD events yielded an O:E of 0.10 (95% CI: 0.05-0.18) in CAC = 0 vs 0.33 (95% CI: 0.27-0.39) in CAC >0 (Supplemental Table 4). Because the PCE predicts hard ASCVD rather than hard CHD alone, the expected count in this supplementary analysis includes stroke events that the observed count does not, resulting in lower O:E ratios than would be obtained with a CHD-specific prediction model. The hard ASCVD O:E ratios above represent the more appropriate calibration comparison.

### Competing risk analysis

Within 10 years, 394 non-CHD deaths occurred. In a Fine-Gray model treating non-CHD death as a competing event, the subdistribution HR for CAC = 0 vs CAC >0 was 0.27 (95% CI: 0.15-0.50), adjusted for age, sex, and race/ethnicity, virtually identical to the cause-specific HR from the primary Cox model (Supplemental Table 6).

### Sensitivity analyses

After excluding participants on lipid-lowering therapy at baseline, results were consistent (N = 1,233; model 1 HR: 0.26; 95% CI: 0.13-0.52), with a significant CAC × age interaction (*P* = 0.0002) (Supplemental Tables 2 and 3).

To evaluate whether demographic imbalances between CAC groups influenced the primary finding, we estimated propensity scores for CAC = 0 status based on age, sex, and race/ethnicity, then excluded participants at the extremes of the propensity distribution where the two groups lacked overlap. In this propensity-trimmed sample (N = 1,406), the model 1 HR was 0.23 (95% CI: 0.12-0.46), consistent with the primary estimate (Supplemental Table 7).

In a sensitivity analysis using the PREVENT-ASCVD equation with a ≥10% threshold to define the high-risk cohort, 1,646 participants (339 CAC = 0, 1,307 CAC >0) with 154 hard CHD events were included. The model 1 adjusted HR was 0.25 (95% CI: 0.13-0.46), and the CAC × age interaction was significant (*P* = 0.0006), with age-specific HRs of 0.24 at age 70, 0.12 at age 75, and 0.06 at age 80 (Supplemental Table 5).

In a sensitivity analysis excluding 263 participants aged ≥80 (16.4% of the cohort; 36 CAC = 0, 227 CAC >0), the model 1 HR was 0.28 (95% CI: 0.15-0.54). The CAC × age interaction was attenuated in this restricted age range (*P* = 0.093), consistent with the removal of the oldest stratum where the protective association was strongest (Supplemental Table 8).

## Discussion

In a well-phenotyped, community-based cohort of adults classified as high risk by the PCE (≥20%), nearly 1 in 4 had CAC = 0 at baseline and experienced substantially fewer hard CHD events over 10 years compared with peers with any CAC. Despite this marked difference in outcomes, individuals with CAC = 0 exhibited broadly similar distributions of major cardiovascular risk factors, including lipids, diabetes status, and blood pressure. Collectively, these findings indicate that within groups designated high risk by conventional calculators, CAC = 0 identifies a subset with comparable traditional risk factor burden but substantially lower observed coronary event rates (Central Illustration).

**Central Illustration:**
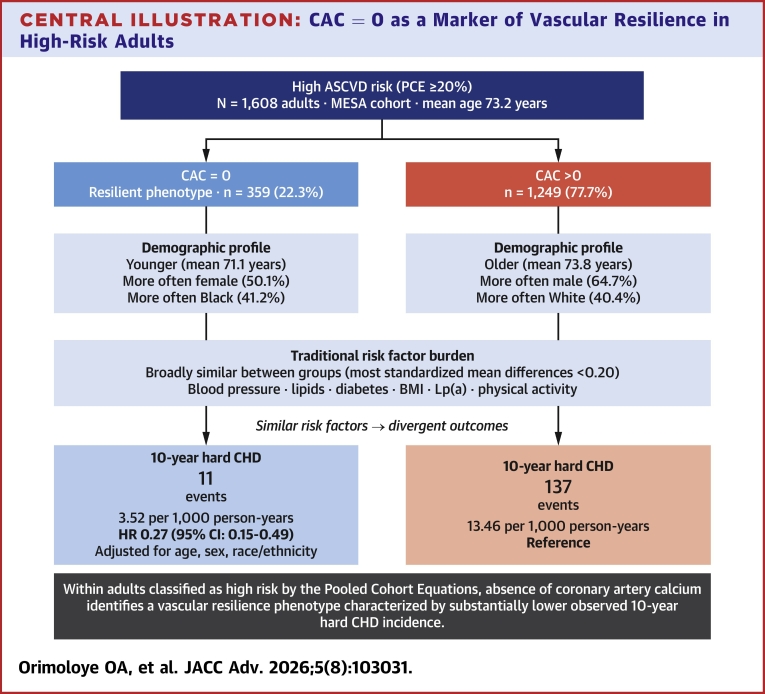
CAC = 0 as a Marker of Vascular Resilience in High-Risk Adults Conceptual representation of the resilient phenotype: among adults classified as high cardiovascular risk by the Pooled Cohort Equations (PCE ≥20%), absence of coronary artery calcium (CAC = 0) identifies a subset with similar traditional risk factor burden but substantially lower observed 10-year hard CHD incidence. HR adjusted for age, sex, and race/ethnicity. BMI = body mass index; other abbreviations as in Figure 1.

As an implication of this study, we are not proposing de-escalatory clinical decisions in this high-risk population, for whom guideline-directed statin therapy remains indicated.^3^ Rather, our framing is discovery-oriented as we propose using CAC to phenotype a subset of high-calculated-risk adults who appear resilient, that is, individuals who, despite a high estimated risk burden, show no detectable calcified atherosclerosis and accrue comparatively few events over 10 years of follow-up. This characterization represents a phenotypic description rather than a mechanistic explanation; identifying the biological pathways that sustain an atherosclerosis-free arterial wall despite prolonged risk factor exposure is an important next step. We believe that the characterization of this group is an important step toward furthering our understanding of protective mechanisms that may be biologically and clinically informative in the years to come.^19^

Our risk findings mirror prior observations in MESA. In an analysis of individuals at the extremes of traditional risk factor burden, Silverman et al^10^ reported that even participants with 3 or more major risk factors had very low CHD event rates when CAC was absent (3.1 per 1,000 person-years over a mean 7-year follow-up). This pattern parallels the present results, where high PCE-risk adults with CAC = 0 also experienced markedly fewer events than peers with any CAC. Extending these observations beyond coronary events, Nasir et al^20^ similarly showed that individuals with multiple risk factors, but no CAC had significantly lower mortality rates relative to individuals without risk factors who had substantial CAC, over a mean follow-up of 5.6 years. Together, these data reinforce the concept that the absence of calcified plaque in high-risk individuals identifies a favorable prognosis despite substantial conventional risk exposure.

The CAC = 0 group demonstrated a modestly higher eGFR than the CAC >0 group (72.2 vs 68.7 mL/min/1.73 m^2^; SMD 0.20). Whether preserved renal function in this context reflects a shared upstream protective process or contributes independently to the resilient phenotype warrants further investigation.

The significant CAC × age interaction, with progressively stronger protective associations at older ages (HR: 0.27 at age 70, 0.18 at age 75, 0.13 at age 80), was consistent across adjustment models and replicated in the PREVENT-defined cohort (*P* = 0.0006). We interpret this pattern as likely reflecting survivor enrichment: among older adults who have maintained CAC = 0 despite decades of risk factor exposure, those reaching advanced age without calcified atherosclerosis represent an increasingly selected group with robust protective biology. The longer the exposure window without plaque development, the stronger the signal of resilience. This interpretation warrants confirmation in larger samples. We note that with only 1 event each in the 70 to 79 and ≥ 80 age strata for CAC = 0, the age-specific HRs in older groups carry substantial imprecision, and the interaction finding should be regarded as hypothesis-generating.

An important alternative hypothesis is that the low event rate in CAC = 0 simply reflects PCE miscalibration rather than true biological resilience. Our O:E calibration analysis addresses this concern directly. While the PCE overestimated risk in both groups, consistent with its known miscalibration in contemporary populations, the degree of overestimation was approximately twice as large in CAC = 0 (O:E 0.26) as in CAC >0 (O:E 0.53), with non-overlapping CIs. Since the PCE does not incorporate CAC information, uniform miscalibration cannot produce differential O:E ratios between CAC groups. The residual gap represents an event deficit specific to CAC = 0 that PCE overestimation alone cannot explain. A recent analysis within MESA confirmed that the PREVENT equation demonstrates superior calibration compared with PCE in this cohort^21^; our sensitivity analysis using PREVENT-ASCVD ≥10% to define the high-risk cohort yielded virtually identical results (model 1 HR: 0.25; 95% CI: 0.13-0.46), confirming that the resilience phenotype is robust to the choice of risk score for cohort definition.

The FH population has been investigated in some prior studies as a window into the idea of resilience, with prevalence estimates of CAC = 0 across these studies ranging from 22% to 69% with a mean pooled prevalence of 45%.^22^ In specific investigations to assess the predictors of “resilient FH” in the SAFEHEART (Spanish Familial Hypercholesterolemia Cohort Study) registry, Pérez de Isla and colleagues identified genetically confirmed FH patients who reached older age without ASCVD to be more often women and characterized by higher HDL-C, lower Lp(a), absence of hypertension, and lower SAFEHEART Risk Equation scores.^23^ Complementing this, Climent et al^24^, using the Spanish Atherosclerosis Society dyslipidemia registry, found that event-free heterozygous familial hypercholesterolemia survivors ≥70 years were more often women, slightly younger, and had fewer comorbidities, with higher HDL-C and lower Lp(a). Conversely, they found clustering of ≥3 conventional risk factors to be strongly associated with ASCVD.^24^

Our findings in this present study are congruent with the reports of Pérez de Isla and Climent with respect to sex and age patterns, but aside from family history and higher cumulative smoking exposure in the CAC >0 group, traditional risk factor burden was broadly similar by CAC status (|SMD|<0.20 for most factors). This pattern argues against risk factors solely dictating atherosclerotic burden even in high-risk groups and reinforces the need to consider biological domains that may sustain an atherosclerosis-free arterial wall despite long exposure windows.

Our methodological approach to the question of resilience differs from those explored in the FH population in several respects. First, it is genotype-agnostic and anchored in a multiethnic community cohort, rather than gene-defined registries. Second, CAC was measured uniformly at baseline, and participants were followed prospectively with adjudicated events over a fixed 10-year horizon, anchoring risk assessment to a standardized exposure measurement and a common analytic time origin. In contrast, registry-based FH studies often infer resilience retrospectively among older survivors with variable timing of enrollment and follow-up. Third, high-risk designation derives from validated risk equations used in routine practice (PCE in the primary analysis and PREVENT in sensitivity analyses), enhancing generalizability beyond specialty FH populations. Together, these distinctions position our study as a parallel but broader operationalization of vascular resilience, extending FH-based insights to the general high-risk population.

We posit that our findings, together with prior studies, suggest that resilience may reflect underlying protective biology rather than simply lower exposure to conventional risk factors. Human genetics provides precedent for this concept.^25^, ^26^, ^27^, ^28^ Protective variants in *PCSK9*, *APOC3*, *ANGPTL3*, and *ASGR1* have been identified in individuals with unusually favorable lipid profiles and lower coronary risk, illustrating how naturally occurring resilience can illuminate disease biology.^25^, ^26^, ^27^ These discoveries later guided or validated preventive therapies, including PCSK9 inhibitors, ANGPTL3 inhibition, and APOC3 antisense approaches.^29^, ^30^, ^31^

Extending this framework, recent polygenic analyses demonstrate that genetic susceptibility remains strongly associated with CAC burden even in adults ≥75 years, independent of traditional risk factors and lifestyle,^32^ suggesting heritable contributions to distinct arterial aging phenotypes that persist across the life course.

A CAC-anchored resilient phenotype could serve a similar purpose by enriching for individuals who harbor protective mechanisms, thereby accelerating discovery of pathways that sustain arterial health. Complementary signals from resilience-oriented genomics, such as reports linking *CETP* variants to event-free aging, together with prior work on protein-based cardiovascular risk models, further support multi-omic profiling of high-risk adults with CAC = 0 as a tractable next step.^33^, ^34^, ^35^

## Study strengths and limitations

Strengths of this study include a prespecified focus on PCE ≥20%, standardized CAC measurement with core-lab adjudication, physician-adjudicated outcomes, a 10-year horizon aligned with clinical risk tools, and consistent results across sensitivity analyses including alternative cohort definition using the PREVENT equations and competing risk modeling. Limitations include single-time CAC assessment at baseline (with the question of the evolution/course of this phenotype reserved for a separate analysis), the relatively small number of hard CHD events in the CAC = 0 group (11 events, with 9 occurring in participants aged <70 and only 1 event each in the 70-79 and ≥ 80 strata), which limits the precision of subgroup and age-stratified analyses, and the potential for residual confounding despite robust adjustment and sensitivity analyses excluding baseline lipid-lowering therapy. Both the PCE and PREVENT equations are validated for ages up to 79 years; 263 participants (16.4%) aged 80 to 84 in this cohort are outside the validated range of both instruments. Survival to enrollment is inherent to this cohort of older adults free of clinical cardiovascular disease, and while it is a structural feature of the study design that enriches for the resilient phenotype, it precludes causal inference about the mechanisms underlying resilience. A full-scale exploration of multiple domains that may contribute to resilience, including genetic, inflammatory, renal, and other biological factors, was beyond the scope of this investigation, which is focused on traditional cardiovascular risk factors.

## Conclusions

Among adults labeled high risk by standard risk estimation, CAC = 0 identifies about 1 in 4 with substantially lower 10-year coronary event rates. We deliberately stop short of treatment implications and instead propose using this phenotype of high calculated ASCVD risk, CAC = 0, and low observed events as an entry point to investigate resilience biology in a scalable way. The next step in this line of inquiry would be to validate this construct within other cohorts and conduct targeted mechanistic studies to identify pathways that could be translated into prevention targets for broader populations.

Perspectives **COMPETENCY IN MEDICAL KNOWLEDGE:** Among adults classified as high risk by the Pooled Cohort Equations (≥20%), nearly 1 in 4 have a coronary artery calcium score of zero and experience substantially lower 10-year hard coronary heart disease event rates than high-risk peers with any detectable calcium, despite comparable traditional risk-factor burden. This event deficit is not fully explained by the known overestimation of risk by the Pooled Cohort Equations, suggesting that absence of calcified atherosclerosis may identify a resilient coronary phenotype warranting mechanistic investigation.

**TRANSLATIONAL OUTLOOK:** Characterizing why some high-risk adults remain free of coronary calcification despite prolonged risk-factor exposure may reveal protective biological mechanisms that could inform new prevention strategies.

## Funding support and author disclosures

This research was supported by contracts 75N92020D00001, HHSN268201500003I, N01-HC-95159, 75N92020D00005, N01-HC-95160, 75N92020D00002, N01-HC-95161, 75N92020D00003, N01-HC-95162, 75N92020D00006, N01-HC-95163, 75N92020D00004, N01-HC-95164, 75N92020D00007, N01-HC-95165, N01-HC-95166, N01-HC-95167, N01-HC-95168, and N01-HC-95169 from the 10.13039/100000050National Heart, Lung, and Blood Institute; and by grants UL1-TR-000040, UL1-TR-001079, and UL1-TR-001420 from the 10.13039/100006108National Center for Advancing Translational Sciences. Dr Orimoloye is supported by the 10.13039/100000050National Heart, Lung, and Blood Institute under award T32HL069771. All other authors have reported that they have no relationships relevant to the contents of this paper to disclose.
