# Supplementary material for: Zero Coronary Artery Calcium as a Marker of Vascular Resilience in High-Risk Adults
Source: JACC Adv. 2026 Jul 20;5(8):103031. doi: 10.1016/j.jacadv.2026.103031 (PMC13393660; doi:10.1016/j.jacadv.2026.103031)
Supplement: Supplemental_Material [file mmc1.pdf]

Supplemental Materials

Zero Coronary Artery Calcium as a Marker of Vascular Resilience in High-Risk Adults: Findings from the MESA Study

Orimoloye OA, Obisesan OH, Osei AD, Oredipe O, Carnethon MR, Blaha MJ, Greenland P, Rasmussen-Torvik LJ

Table S1. Fully adjusted Cox model (Model 2) for CAC=0 vs CAC>0 and 10-year hard CHD events

**Variable**	**HR (95% CI)**	**P value**
**CAC = 0 (vs CAC > 0)**	0.32 (0.18–0.59)	<0.001
Age (per year)	1.03 (0.99–1.07)	0.114
Male sex	1.70 (1.13–2.54)	0.010
Race/ethnicity (ref: White)		
Chinese	0.80 (0.40–1.61)	0.538
Black	1.14 (0.71–1.82)	0.598
Hispanic	1.49 (0.97–2.31)	0.069
SBP (per mmHg)	1.01 (1.00–1.01)	0.118
LDL-C (per mg/dL)	1.00 (1.00–1.01)	0.509
Smoking (ref: Never)		
Former	0.75 (0.50–1.11)	0.154
Current	0.84 (0.45–1.59)	0.602
Diabetes (ref: Normal)		
IFG	0.73 (0.42–1.26)	0.256
Untreated DM	0.93 (0.37–2.32)	0.881
Treated DM	1.32 (0.85–2.06)	0.212
Family history of CHD	1.16 (0.80–1.67)	0.436

N = 1,431. Events: 128. Diabetes categorized as normal, IFG, untreated DM, or treated DM.

Table S2. Baseline characteristics, sensitivity cohort (excluding baseline lipid-lowering therapy)

Variable	CAC = 0 (n=291)	CAC > 0 (n=942)	SMD
**Sociodemographic**			
Age (years)	71.3 (7.3)	73.9 (6.3)	**0.39**
Male (%)	50.2	67.2	**0.35**
Race/ethnicity (%)			
White	19.2	40.3	**0.47**
Black	39.9	25.0	**0.32**
Chinese	12.4	11.2	0.04
Hispanic	28.5	23.6	0.11
Education > high school (%)	48.8	54.1	0.11
Income > $50,000 (%)	20.9	23.9	0.07
**Clinical risk factors**			
SBP (mmHg)	146.1 (21.5)	143.5 (21.3)	0.12
DBP (mmHg)	76.3 (11.7)	74.3 (10.5)	0.17
Total cholesterol (mg/dL)	194.8 (35.4)	196.8 (37.5)	0.06
Triglycerides (mg/dL)*	114 [75–170]	120 [85–169]	0.01
LDL-C (mg/dL)	117.1 (30.3)	121.2 (31.6)	0.13
HDL-C (mg/dL)	50.1 (14.4)	48.1 (14.5)	0.14
Lp(a) >50 mg/dL (%)	19.9	19.8	0.00
Fasting glucose (mg/dL)*	98 [89–124]	95 [87–110]	0.16
BMI (kg/m²)	28.6 (5.4)	27.9 (4.9)	0.13
eGFR, CKD-EPI (mL/min/1.73 m²)	72.9 (16.9)	69.4 (17.0)	**0.20**
Diabetes (%)	35.7	24.8	**0.24**
Hypertension (%)	80.1	74.1	0.14
Family history of CHD (%)	36.0	45.7	**0.20**
Smoking status (%)			
Never	49.8	43.3	0.13
Former	33.7	42.0	0.17
Current	16.5	14.7	0.05
Pack-years*	0.0 [0.0–14.5]	3.0 [0.0–26.0]	**0.32**
Exercise ≥500 MET-min/wk (%)	57.2	61.3	0.08

N = 1,233. Bold SMDs denote |SMD| ≥0.20. Asterisk (*) denotes median [IQR].

Table S3. Cox regression results, sensitivity cohort (excluding baseline lipid-lowering therapy)

Model	HR (95% CI)	P value
Unadjusted	0.24 (0.12–0.49)	<0.001
Model 1 (age, sex, race/ethnicity)	0.26 (0.13–0.52)	<0.001
Model 2 (fully adjusted)	0.31 (0.15–0.64)	0.002
**Effect modification**		
CAC × age interaction (Model 1)		0.0002
HR at age 70	0.27 (0.13–0.59)	0.001
HR at age 75	0.16 (0.07–0.35)	<0.001
HR at age 80	0.09 (0.04–0.23)	<0.001

N = 1,233 (Model 1); N = 1,086 (Model 2). Events: 107/92. Lipid-lowering therapy excluded.

Table S4. Observed-to-expected (O:E) ratios for PCE-predicted events by CAC status

Endpoint	Group	N	Observed	Expected	O:E	95% CI
Hard ASCVD	CAC = 0	359	28	108.2	0.26	0.17–0.37
Hard ASCVD	CAC > 0	1,249	222	419.1	0.53	0.46–0.60
Hard CHD	CAC = 0	359	11	108.2	0.10	0.05–0.18
Hard CHD	CAC > 0	1,249	137	419.1	0.33	0.27–0.39

Expected = sum of PCE-predicted ASCVD probabilities. 95% CIs: exact Poisson. CHD O:E ratios are lower than would be obtained with a CHD-specific prediction model because the expected count includes stroke events that the observed count does not.

Table S5. Sensitivity analysis using PREVENT-ASCVD ≥10% for cohort definition

Panel A: Baseline characteristics (PREVENT cohort)

Variable	CAC = 0 (n=339)	CAC > 0 (n=1,307)	SMD
**Sociodemographic**			
Age (years)	72.3 (6.6)	74.2 (6.1)	**0.30**
Male (%)	45.7	61.9	**0.33**
Race/ethnicity (%)			
White	21.2	39.7	**0.41**
Black	40.7	26.7	**0.30**
Chinese	13.3	11.6	0.05
Hispanic	24.8	22.0	0.07
Education > high school (%)	51.0	52.8	0.04
Income > $50,000 (%)	18.7	23.7	0.12
**Clinical risk factors**			
SBP (mmHg)	143.8 (24.5)	140.9 (23.5)	0.12
DBP (mmHg)	74.8 (11.8)	72.7 (10.7)	0.19
Total cholesterol (mg/dL)	190.6 (36.7)	194.2 (38.0)	0.10
Triglycerides (mg/dL)*	114 [77–170]	122 [84–173]	0.04
LDL-C (mg/dL)	114.4 (32.2)	118.4 (31.7)	0.12
HDL-C (mg/dL)	48.9 (12.9)	47.7 (13.5)	0.09
Lp(a) >50 mg/dL (%)	18.8	20.5	0.04
Fasting glucose (mg/dL)*	98 [88–122]	95 [87–113]	0.09
BMI (kg/m²)	28.7 (5.6)	28.1 (4.9)	0.11
eGFR, CKD-EPI (mL/min/1.73 m²)	69.5 (17.2)	67.7 (17.4)	0.10
Diabetes (%)	36.6	27.9	0.19
Hypertension (%)	79.4	73.9	0.13
Lipid-lowering therapy (%)	15.3	22.7	0.19
Family history of CHD (%)	36.8	46.4	0.19
Smoking status (%)			
Never	54.0	45.8	0.16
Former	31.3	41.8	**0.22**
Current	14.8	12.5	0.07
Pack-years*	0.0 [0.0–13.0]	1.8 [0.0–24.6]	**0.25**
Exercise ≥500 MET-min/wk (%)	56.8	60.8	0.08
PREVENT-ASCVD risk (%)	13.8 (3.6)	14.8 (3.8)	**0.29**

Panel B: Cox regression results (PREVENT cohort)

**Model**	**HR (95% CI)**	**P value**
Unadjusted	0.25 (0.13–0.47)	<0.001
Model 1 (age, sex, race/ethnicity)	0.25 (0.13–0.46)	<0.001
Model 2 (fully adjusted)	0.30 (0.16–0.57)	<0.001
**Effect modification**		
CAC × age interaction (Model 1)		0.0006
HR at age 70	0.24 (0.12–0.51)	<0.001
HR at age 75	0.12 (0.05–0.31)	<0.001
HR at age 80	0.06 (0.02–0.21)	<0.001

N = 1,646 (339 CAC=0, 1,307 CAC>0). Events: 154 (10/144). PREVENT-ASCVD ≥10% per 2026 ACC/AHA guidelines.

Table S6. Competing risk analysis: cause-specific vs subdistribution hazard ratios

Model	Estimate (95% CI)	P value
Cause-specific HR (standard Cox)	0.27 (0.15–0.49)	<0.001
Subdistribution HR (Fine-Gray)	0.27 (0.15–0.50)	<0.001

Both adjusted for age, sex, race/ethnicity. N = 1,608. CHD events: 148. Non-CHD deaths: 394 within 10 years.

Table S7. Propensity-trimmed sensitivity analysis

Model	HR (95% CI)	P value
Unadjusted	0.24 (0.12–0.47)	<0.001
Model 1 (age, sex, race/ethnicity)	0.23 (0.12–0.46)	<0.001

Propensity scores for CAC=0 status estimated using logistic regression with age, sex, and race/ethnicity. Analysis restricted to participants within the 5th to 95th percentile of the CAC=0 propensity score distribution. N = 1,406 (328 CAC=0, 1,078 CAC>0). Events: 126 (9 CAC=0, 117 CAC>0). Trimming bounds: propensity score 0.47–0.91.

Table S8. Sensitivity analysis excluding participants aged ≥80

Model	HR (95% CI)	P value
Unadjusted	0.30 (0.16–0.57)	<0.001
Model 1 (age, sex, race/ethnicity)	0.28 (0.15–0.54)	<0.001
**Effect modification**		
CAC × age interaction (Model 1)		0.093
HR at age 70	0.26 (0.13–0.50)	<0.001
HR at age 75	0.20 (0.09–0.43)	<0.001

263 participants (16.4%) aged ≥80 excluded (36 CAC=0, 227 CAC>0). Remaining N = 1,345 (323 CAC=0, 1,022 CAC>0). Events: 110 (10 CAC=0, 100 CAC>0). The attenuated age interaction (p = 0.093 vs. 0.023 in the full cohort) is consistent with the removal of the ≥80 stratum where the protective association was strongest.

Supplemental Figure Legends

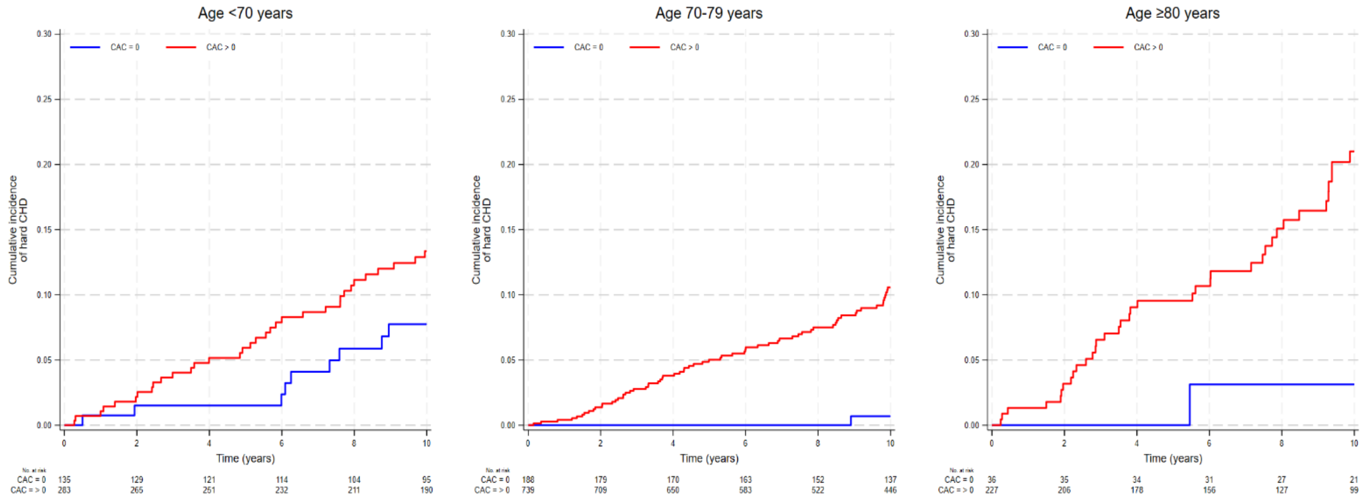

Figure S1. Age-stratified Kaplan–Meier cumulative incidence of hard CHD events by CAC status among adults with PCE ≥20% (N = 1,608). Panels show participants aged <70 years (left), 70–79 years (center), and ≥80 years (right). Hard CHD event counts in the CAC = 0 group were 9 (<70 years), 1 (70–79 years), and 1 (≥80 years). The near-zero cumulative incidence in older CAC = 0 strata is consistent with the significant CAC × age interaction ($p = 0.023$) but should be interpreted with caution given sparse events.

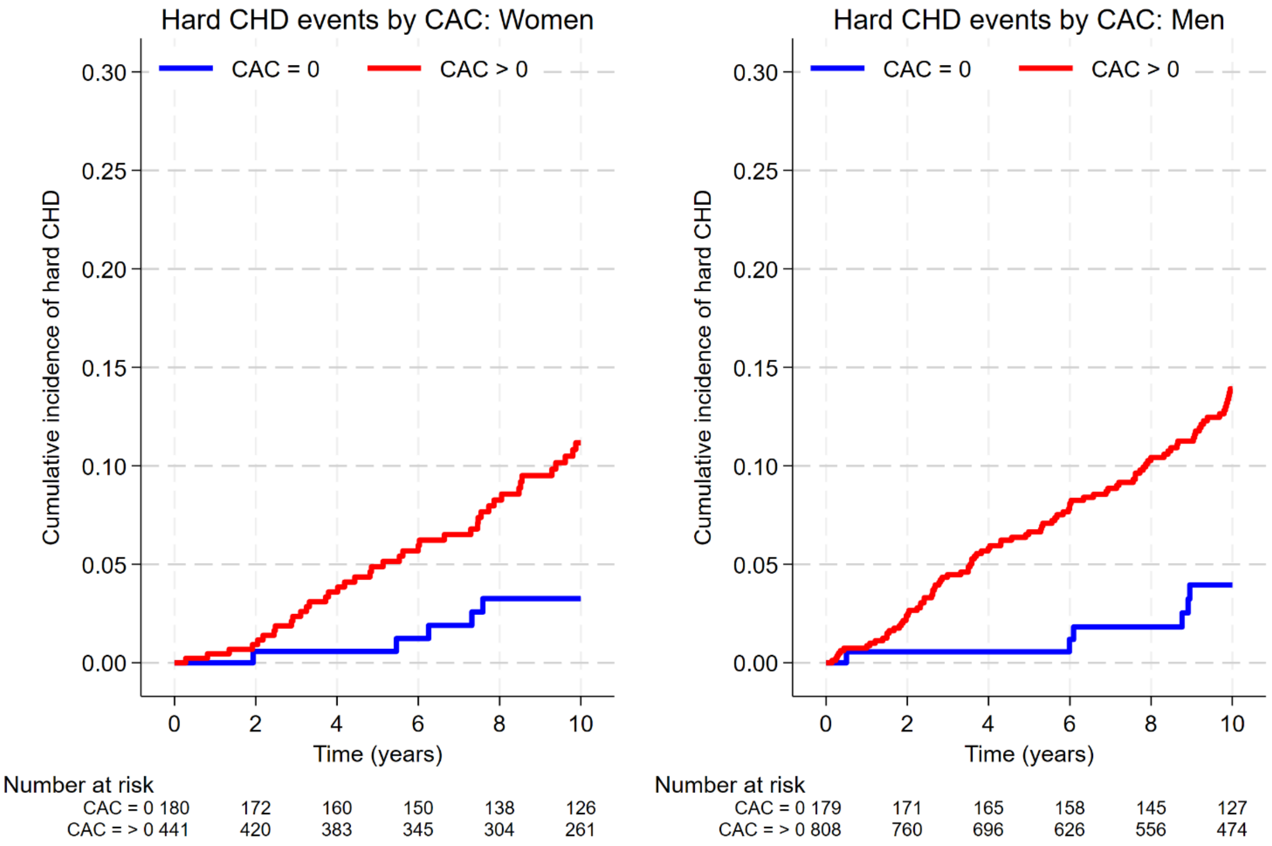

Figure S2. Sex-stratified Kaplan–Meier cumulative incidence of hard CHD events by CAC status among adults with PCE ≥20% (N = 1,608). Panels show women (left) and men (right). No significant CAC × sex interaction was observed ($p = 0.89$).

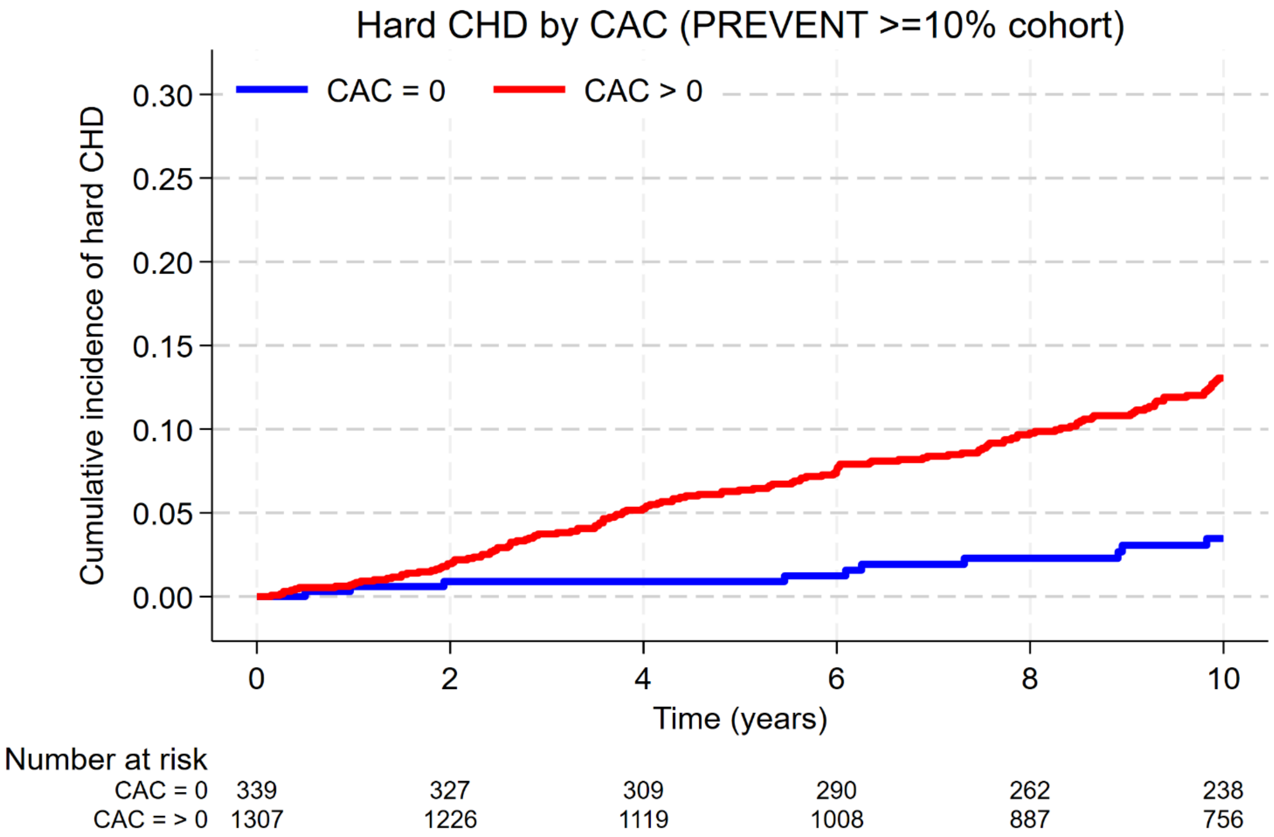

Figure S3. Kaplan–Meier cumulative incidence of hard CHD events by CAC status in the PREVENT-defined high-risk cohort (PREVENT-ASCVD ≥10%; N = 1,646). Log-rank $p < 0.001$.
